# Lane Detection Algorithm in Curves Based on Multi-Sensor Fusion

**DOI:** 10.3390/s23125751

**Published:** 2023-06-20

**Authors:** Qiang Zhang, Jianze Liu, Xuedong Jiang

**Affiliations:** School of Mechanical and Automotive Engineering, Qingdao University of Technology, Qingdao 266520, China; zhangqiang@qut.edu.cn (Q.Z.); jiangxuedong98@163.com (X.J.)

**Keywords:** lane line identification, sensor fusion, sliding window, curves with large curvature

## Abstract

Identifying lane markings is a key technology in assisted driving and autonomous driving. The traditional sliding window lane detection algorithm has good detection performance in straight lanes and curves with small curvature, but its detection and tracking performance is poor in curves with larger curvature. Large curvature curves are common scenes in traffic roads. Therefore, in response to the problem of poor lane detection performance of traditional sliding window lane detection algorithms in large curvature curves, this article improves the traditional sliding window algorithm and proposes a sliding window lane detection calculation method, which integrates steering wheel angle sensors and binocular cameras. When a vehicle first enters a bend, the curvature of the bend is not significant. Traditional sliding window algorithms can effectively detect the lane line of the bend and provide angle input to the steering wheel, enabling the vehicle to travel along the lane line. However, as the curvature of the curve increases, traditional sliding window lane detection algorithms cannot track lane lines well. Considering that the steering wheel angle of the car does not change much during the adjacent sampling time of the video, the steering wheel angle of the previous frame can be used as input for the lane detection algorithm of the next frame. By using the steering wheel angle information, the search center of each sliding window can be predicted. If the number of white pixels within the rectangular range centered around the search center is greater than the threshold, the average of the horizontal coordinate values of these white pixels will be used as the horizontal coordinate value of the sliding window center. Otherwise, the search center will be used as the center of the sliding window. A binocular camera is used to assist in locating the position of the first sliding window. The simulation and experimental results show that compared with traditional sliding window lane detection algorithms, the improved algorithm can better recognize and track lane lines with large curvature in bends.

## 1. Introduction

In today’s era, cars have become an indispensable means of transportation. The increasingly complex traffic conditions have made advanced driving assistance systems (ADAS) an essential tool for people to safely drive and travel. Lane detection is a key and challenging part of ADAS. Most existing lane detection algorithms focus on detecting straight lane lines. Li et al. used Hough transform and Canny edge detection to detect straight lanes [1]. Some scholars have also studied and improved Hough transform to achieve higher computational speed and better detection results [2,3,4]. Hale et al. proposed a hybrid method to improve the efficiency of lane detection [5], which has high anti-interference and detection efficiency. With the increasingly widespread application of machine learning and deep learning, many intelligent driving researchers have also added machine-learning algorithms to lane detection [6,7,8,9]. Sayyidul et al. added clustering algorithms to lane detection algorithms [10], making them more accurate compared to traditional lane detection algorithms. Although the lane line detection algorithms proposed above have certain advantages, most of them only focus on lane line detection for straight lanes, with limited research on curved lanes, thus having certain limitations. Curves are a common part of road traffic systems and are also areas prone to traffic accidents. How to detect lane lines in curves and provide traffic information to drivers in a timely manner is also an important part of assisting safe driving.

Many scholars have also studied the detection of curved lanes. Wang et al. proposed a curve detection algorithm based on a straight curve model [11]. The simulation and experimental results indicate that this method has good applicability for most curved road conditions. However, this algorithm is targeted at small curvature curves on highways or urban roads, with a radius of generally over 600 m. It is not suitable for some large curvature curves on urban roads or ramps at highway intersections. Li et al. proposed a B-spline curve fitting method based on binary particle swarm optimization [12]. Although it has strong applicability, it is only suitable for curves and solid lanes with a large radius, and the control points will be lost in the dotted lane, which will reduce the accuracy of fitting. Sun and He [13] proposed the method of using sliding windows to detect lane lines. This algorithm has good performance in curves with small curvature, but when the curvature of the curve is large or the lane line is dashed, some sliding windows may not track the lane line, making the fitting effect of the lane line unreasonable. Chen et al. proposed a detection method for sharp bends [14], and experimental results showed that their proposed model had high accuracy in detection, but the detection effect was not ideal in curves with significant curvature changes. Wang et al. proposed a multi-step curve lane detection algorithm [15], which mainly includes two parts: one is the parallel straight line model, and the other is the Hyperbola model. Although its experimental results indicate that the algorithm has good robustness in noisy environments, it targets curves with smaller curvature and has not been discussed or experimented with curves with larger curvature. Therefore, the scene it targets has certain limitations. Wei et al. proposed a linear curve automatic detection and classification method based on Gist SVM machine learning [16]. The experimental results show that their proposed method can reduce the error detection rate of curved lane lines by 20% to improve detection accuracy. However, using machine-learning methods to detect curved lane lines requires a large amount of lane line data, which need to be manually obtained in advance, which will consume a lot of manpower and material resources. Additionally, a 20% reduction in detection error rate is only an average, and the detection error rate in curves with larger curvature will be significantly higher than in other situations. Zhang et al. proposed a curved lane detection method based on lane projection [17], which utilizes morphology to extract lane information. The experimental results show that this method can effectively detect lane lines in solid curves with high curvature, but the detection effect is not ideal for dashed lane lines with large curvature.

In view of the above situation, this paper proposes a sliding window detection lane line algorithm, which combines the steering wheel angle sensor and binocular camera. This algorithm has two more important parts: one is to use the steering wheel angle information at the previous moment to calculate the radius of the current curve where the vehicle is located, and the other is to calculate the ratio of the pixel distance in the aerial view coordinate system to the real distance in the world coordinate system. The main process of the algorithm is as follows. Firstly, the position of the first sliding window in the aerial view is determined by pixel statistics. If the white pixels detected by pixel statistics are less than the threshold value, the position of the first sliding window is determined by binocular camera ranging. Secondly, use the angle information of the steering wheel from the previous moment to calculate the radius of the curve where the vehicle is located at the current moment; calculate the offset of the search center of each sliding window relative to the center of the first sliding window using the distance ratio method, and search for white pixels within a rectangular range centered on the search center. If the number of pixels is greater than the threshold, the average of the horizontal coordinates of these white pixels will be used as the horizontal coordinate value of the current window center point. Otherwise, the search center will be used as the window center point. Finally, the center point of the sliding window is fitted using the least squares method to obtain the final lane line.

## 2. Implementation Methods

This section mainly introduces how to calculate the turning radius of the vehicle and how to match the pixel distance in the aerial view with the real distance in the world coordinate system. At the same time, we will also introduce how traditional sliding window algorithms detect lane lines.

### 2.1. Calculation of Vehicle Turning Radius

**Figure 1:**
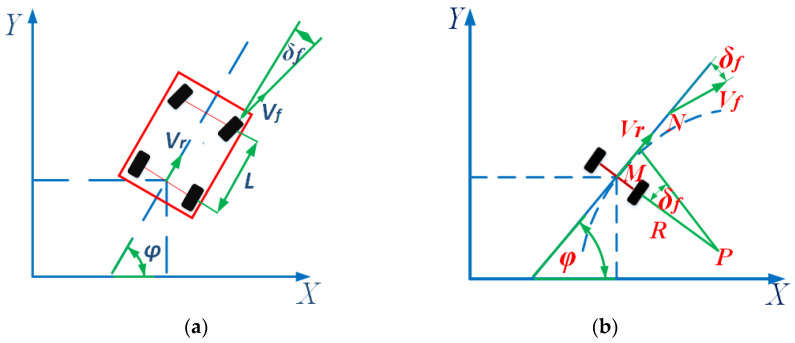
Vehicle motion model diagram and schematic diagram of vehicle front wheel steering: (**a**) Vehicle motion model diagram; (**b**) Schematic diagram of vehicle front wheel steering.

The vehicle motion model is shown in Figure 1a. In the inertial coordinate system *OXY*, φ is the yaw angle (heading angle) of the vehicle body, $\delta_{f}$ is the deflection angle of the front wheel, $v_{r}$ is the center speed of the rear axle of the vehicle, $v_{f}$ is the center speed of the vehicle’s front axle, and *l* is the wheelbase. Figure 1b shows a schematic diagram of the vehicle’s steering process, where *R* represents the rear wheel turning radius, *P* represents the instantaneous center of rotation of the vehicle, *M* represents the rear axle axis of the vehicle, and *N* represents the front axle axis. Assuming that the sideslip angle of the vehicle’s center of mass remains constant during the turning process, the instantaneous turning radius of the vehicle is the same as the curvature radius of the road. The calculation formula for the vehicle’s yaw rate is shown in Formula (1):

(1) $$\omega =\frac{v_{r}}{l}\tan\delta_{f}$$

In Formula (1), $v_{r}$ is the speed at the rear axle axis of the vehicle. The turning radius of the vehicle is shown in Formula (2):

(2) $$R=\frac{v_{r}}{\omega }$$

Substituting the vehicle’s yaw rate into Formula (2) yields Formula (3). The front wheel angle $\delta_{f}$ in Formula (3) can be obtained through the steering wheel angle and steering ratio.

(3) $$R=\frac{l}{\tan\delta_{f}}$$

The calculation of the vehicle turning radius given in Formula (3) is only applicable to vehicles at lower speeds (v ≤ 10 km/h). When the vehicle speed is high, the turning radius of the vehicle will be affected by the tire sideslip angle. At this time, the turning radius of the vehicle is shown in Formula (4).

(4) $$R=(1+Ku^{2})R_{0}$$

In the above formula, *K* is the stability factor, $R_{0}$ is the turning radius of the vehicle at low speeds, and the calculation method for $R_{0}$ is shown in Formula (3). The calculation of stability factor *K* is shown in Formula (5).

(5) $$K=\frac{m}{l^{2}}(\frac{a}{k_{2}}-\frac{b}{k_{1}})$$

In Formula (5), *a* is the distance from the front axle of the vehicle to the center of mass, *b* is the distance from the rear axle of the vehicle to the center of mass, $k_{1}$ is the total lateral stiffness of the front wheels of the vehicle, $k_{2}$ is the total lateral stiffness of the rear wheels of the vehicle, *m* is the body mass, and *l* is the wheelbase. By using Formulae (3)–(5), the turning radius of a vehicle at high speeds can be determined.

### 2.2. Sliding Window Recognition of Lane Lines

In order to better obtain the curvature of the lane line, we need to transform the image taken by the camera into an aerial view through perspective transformation. The advantage of this is that it is easy to detect and improve the detection accuracy.

The principle of perspective transformation [18,19] is shown in Formula (6).

(6) $$\left[\begin{array}{ccc} x^{\prime } & y^{\prime } & w^{\prime } \end{array}\right]=\left[\begin{array}{ccc} u & v & w \end{array}\right]\left[\begin{array}{ccc} a_{11} & a_{12} & a_{13}\\ a_{21} & a_{22} & a_{23}\\ a_{31} & a_{32} & a_{33} \end{array}\right]$$

In Formula (6), (*u*, *v*) are the original image pixel coordinates, (*x*, *y*) are the transformed image pixel coordinates, $x=\frac{x^{\prime }}{w^{\prime }}$, $y=\frac{y^{\prime }}{w^{\prime }}$. Because we are dealing with two-dimensional images, we can set $w^{\prime }=1$. Therefore, to complete the perspective change, the pixel coordinate values of four corresponding points are required.

**Figure 2:**
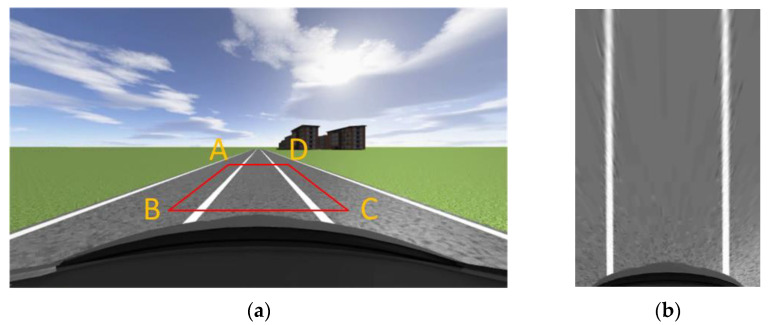
Normal view road map and bird’s eye view road map: (**a**) ROI region selection; (**b**) Aerial view of the driveway.

In order to better detect lane lines in the lane where the vehicle is located, it is also necessary to select ROI regions in the image. Unlike general ROI region selection, in this article, the selection of lane ROI regions not only needs to extract a single lane, but it also needs to make the boundaries of the ROI region as level as possible with the lane line. The advantage of doing so is that the lane in the bird’s eye view can be closer to the lane situation in world coordinates, keeping the error as small as possible during distance matching. In order to obtain a more accurate top view of the lane, we conducted a simulation in Prescan. Figure 2a shows the normal perspective road map taken by the camera before the perspective change. The width of the image is 640, the height is 360, and the red box represents the selected ROI area. The pixel coordinates of each point in ABCD are shown in Table 1. After the perspective change, points *A*, *B*, *C*, and *D* are transformed into *a*(0,0), *b*(0,360), *c*(240,360), and *d*(240,0). Figure 2b shows the road surface after perspective changes. The width and height of the image after perspective transformation are 240, 360.

**Figure 3:**
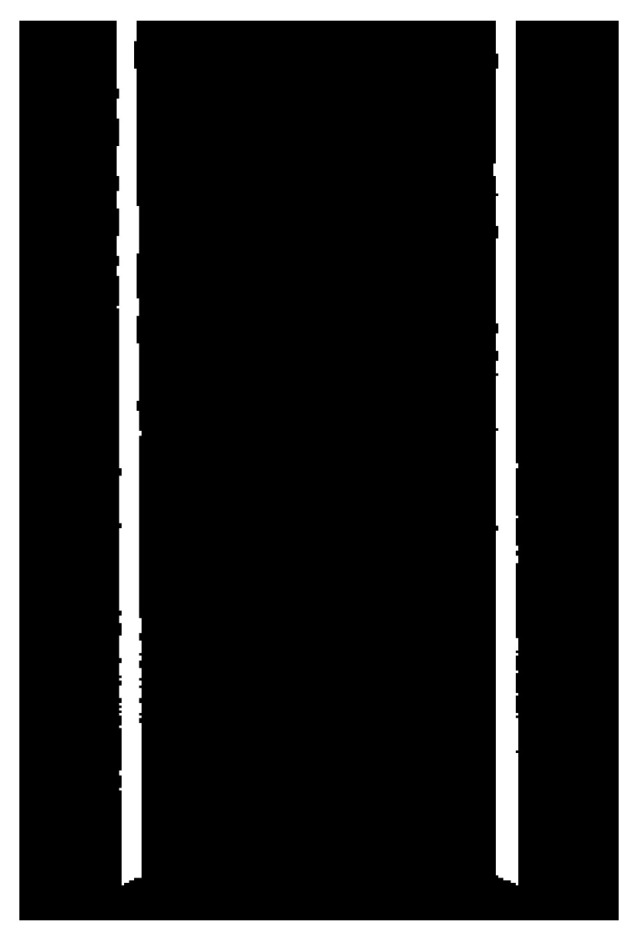
Binary diagram of aerial view.

As can be seen from Figure 2b, when the car is traveling in a straight line, the two lane lines in the aerial view are basically parallel, close to the top view in the world coordinate system. This indicates the rationality of selecting various points in the ROI area and provides a guarantee for the subsequent calculation of the curvature of the lane line. After obtaining the aerial view, the image should be binarized. This is because the original image obtained by the camera is a color image, which has a large amount of data and contains a lot of interference information. Converting the image into a binary image can not only effectively remove this interference information but also improve the operation speed. Moreover, through threshold segmentation, the lane line can be better distinguished from the road surface, and the lane line can be recognized better and faster. The binary diagram of the aerial view of the pavement is shown in Figure 3.

**Figure 4:**
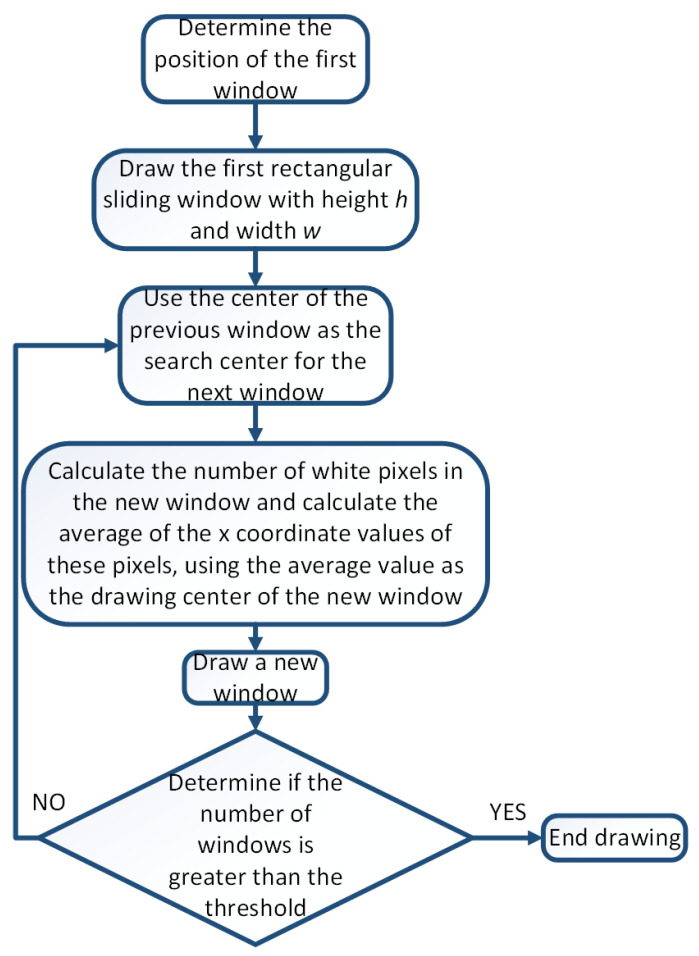
Flow chart of traditional sliding window detection of lane lines.

After preprocessing the image, the sliding window method can be used to detect lane lines [20]. The flow chart of traditional sliding window detection of lane lines is shown in Figure 4.

**Figure 5:**
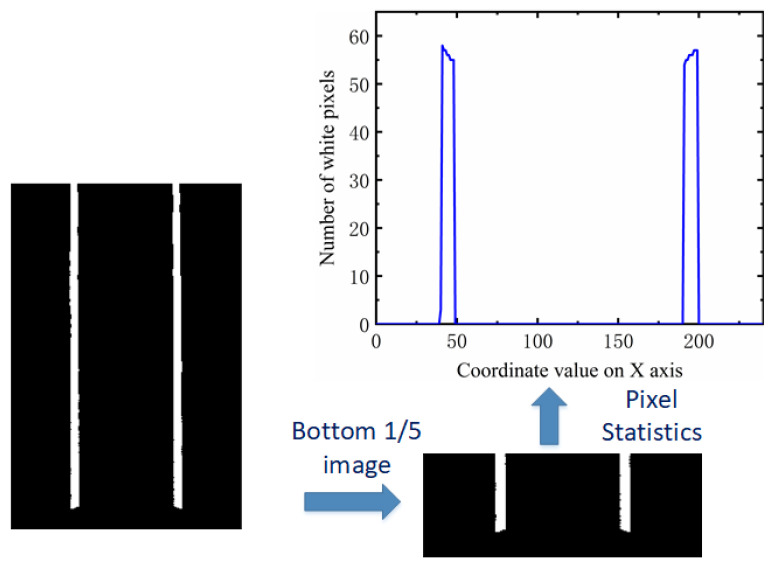
Bottom image and pixel statistics results.

It is very important to determine the coordinate value of the search center of the first window in the sliding window algorithm. This article uses pixel statistics to determine the search center position of the first window. Binocular cameras are used to assist in locating the position of the first sliding window. We define the first window at the bottom of the image, with a width and height of h. We showed that the width and height of the converted aerial view are 240 and 360, respectively. Therefore, it can be determined that the y-coordinate value of the first window center point is 360-h/2, and what needs to be determined is the x-coordinate value of the window center. By calculating the number of white pixels in each column and finding the two columns with the highest number of white pixels, the x-coordinate value of the search center of the first sliding window of the left and right lane lines can be determined. This is the pixel statistics method. When the vehicle is in a curve, the lane line is also curved. If white pixel statistics are carried out on the whole aerial view image, the accuracy of the statistical results will be affected. Therefore, we intercept 1/5 of the aerial view image from the bottom to count the number of white pixels. As shown in Figure 5, it is a pixel statistical map at a certain time.

**Figure 6:**
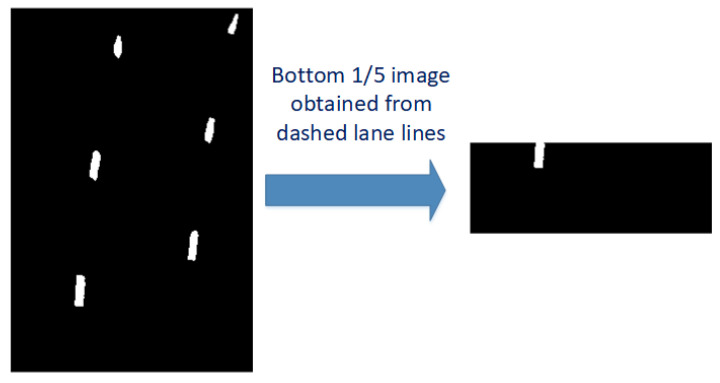
Bottom 1/5 image of dashed lane line.

For most cases, the pixel statistics method can be used to determine the position of the first sliding window of the left and right lane lines. However, for some dashed lane lines, as shown in Figure 6, the right lane line disappears after taking the bottom 1/5 of the image. The pixel statistics method may fail due to the inability to detect white pixels, so a binocular camera is needed to assist in determining the position of the first sliding window. How to use binocular cameras to assist in locating the position of the first sliding window will be mentioned later.

**Figure 7:**
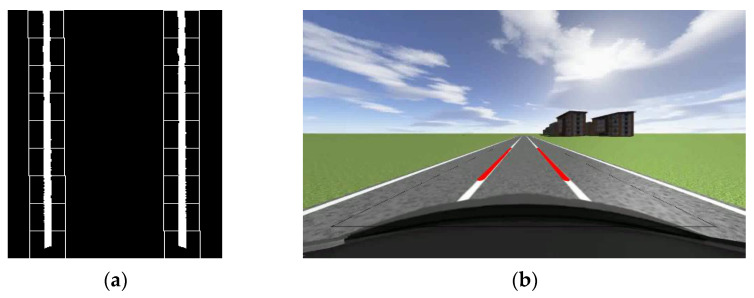
Sliding window diagram and lane line fitting diagram: (**a**) The tracking effect of sliding window on lane lines; (**b**) Lane line fitting diagram.

From Figure 5, it can be seen that there are two obvious peaks in the statistical value of the number of white pixel points. The x-coordinate values of these two peaks can be used as the search center x-coordinate values for the first sliding window of the left and right lane lines. After determining the search center, one can draw a virtual rectangle with a width of *w* and a height of *h*. The values of *w* and *h* can be determined based on the image size. In this article, the values of *w* and *h* are both 40-pixel values. Calculate the sum of the x-coordinate values of all white pixels within the virtual rectangle range and the number of white pixels, and take the average value. This average value is the x-coordinate value of the center of the actual sliding window drawn. The center of the previous sliding window serves as the search center for the next window. Calculate the total number of white pixels and synthesis of the x-coordinate values of these white pixels in the new window, and calculate their mean. Use the mean as the horizontal coordinate value of the new sliding window center and draw a new sliding window. Repeat the above operation until the number of sliding windows exceeds the threshold. The center of the sliding window is fitted with a curve, and then, the aerial view is inverse perspective transformed. The final lane line fitting effect can be obtained. The traditional sliding window effect is shown in Figure 7a, and the fitting of lane lines is shown in Figure 7b.

**Figure 8:**
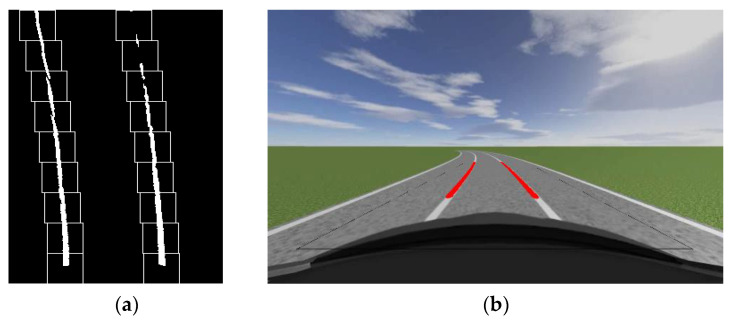
Detection results of traditional sliding window algorithm in small curvature curves: (**a**) The tracking effect of sliding window on lane lines; (**b**) Lane line fitting diagram.

From Figure 7, it can be seen that the lane lines detected and fitted using sliding windows have a good effect. Compared to the method of detecting lane lines using Hough transform, the method of detecting lane lines using sliding windows has better detection performance and better robustness in curves with small curvature. Figure 8 shows the lane detection results of the sliding window for small curvature curves. The curvature radius of the curve is 120 m. From Figure 8, we can also see that when the vehicle first enters a curve (at which point the curvature of the curve is small), the traditional sliding window algorithm can detect the lane line, thereby providing a steering wheel angle control, which can be used as input for subsequent algorithms.

**Figure 9:**
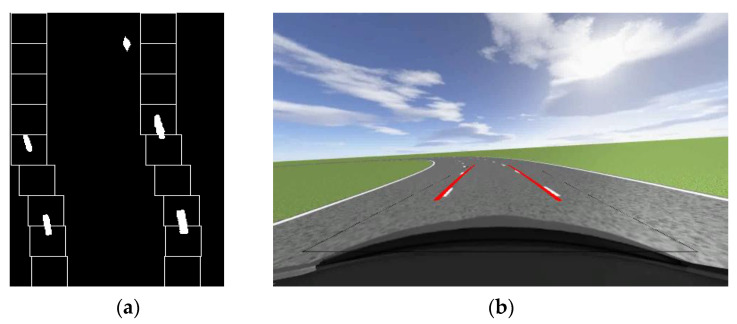
Detection results of traditional sliding window algorithm in dashed curves: (**a**) The tracking effect of sliding window on lane lines; (**b**) Lane line fitting diagram.

Although traditional sliding window algorithms have good detection performance for small curvature curves, in curves with larger curvature or curves with dashed lane lines, some sliding windows may miss or fail to detect white pixels, as shown in Figure 9. The radius of the bend in Figure 9 is 40 m. At this point, the lane lines detected by the traditional sliding window algorithm will have a significant difference from the actual situation. This is disadvantageous in an auto drive system or auxiliary driving system.

### 2.3. Distance Matching

**Figure 10:**
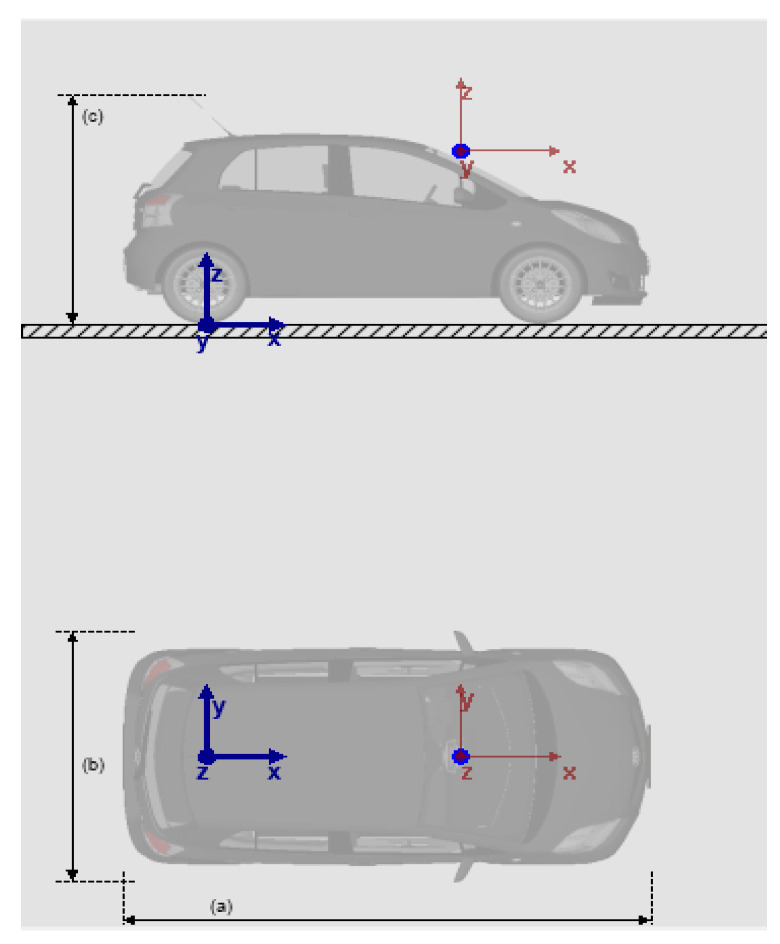
Camera positioning diagram.

The turning radius of a vehicle can be obtained by Formulae (3)–(5), which can also be approximated as the radius of the curve where the vehicle is located. By using the radius, we can calculate the lateral distance difference and longitudinal distance difference between two points on the lane. However, this difference is the true value in the world coordinate system, which is not applicable in the pixel coordinates. Therefore, we still need to calculate the pixel distance difference in the aerial view (including the difference between the horizontal distance and the vertical distance). It is worth noting that both the transverse and longitudinal directions we mentioned are based on the vehicle body as a reference. The distance parallel to the longitudinal axis of the vehicle is called the longitudinal distance, while the distance perpendicular to the longitudinal axis of the vehicle is called the transverse distance. As mentioned earlier, when making changes to the aerial view of the image, we make the lane lines when driving in a straight line as parallel as possible in the aerial view, which will make the aerial view obtained through perspective changes closer to the real-world aerial view. This article proposes a ratio method to calculate the ratio of pixel distance in the image coordinate system to the distance in the world coordinate system. The ratio method can be seen as a calibration process. First, we need to determine the installation position of the camera. Second, we need to determine the transformation matrix of the aerial view, which is related to the four points selected for perspective transformation. Therefore, once these parameters change, we need to regain the scale relationship. Generally speaking, the position of the camera will not move after installation, and the selection of perspective transformation points will not change, so this method is feasible. The reference origin and reference coordinate system of the camera are shown in the blue coordinate system in Figure 10. In the simulation software, the coordinates of x, y, and z are 1.85, 0, 1.4, in meters. The red coordinate system is the camera coordinate system, and its origin is the camera. We take pictures of lanes with different widths and calculate the pixel distance of these lanes in the aerial view, so as to obtain the search scale relationship. There are two main reasons why we use ratio calculation instead of coordinate transformation: the first is that the ratio method can simplify the calculation steps, shorten the calculation time, and obtain the calculation results faster, ensuring real time and effectiveness; the second reason is that using the coordinate transformation method first requires determining the world coordinate system and its origin, and the world coordinate system changes accordingly during the vehicle’s movement, which increases our computational difficulty. Therefore, considering the above two points, we adopt the ratio method. The distance ratio results obtained in the simulation software are shown in Table 2.

The average value of the ratio calculated from Table 2 is 44.7, which means that the distance of one meter in the horizontal position of the world coordinate system in the aerial view image is 44.7 pixels. The ratio of longitudinal distance is shown in Table 3.

The average value of the ratio calculated from Table 3 is 30.8, which means that the distance of one meter from the longitudinal position in the world coordinate system in the aerial view image is 30.8 pixels.

## 3. Sliding Window Algorithm for Fusion of Steering Wheel Angle Sensor

**Figure 11:**
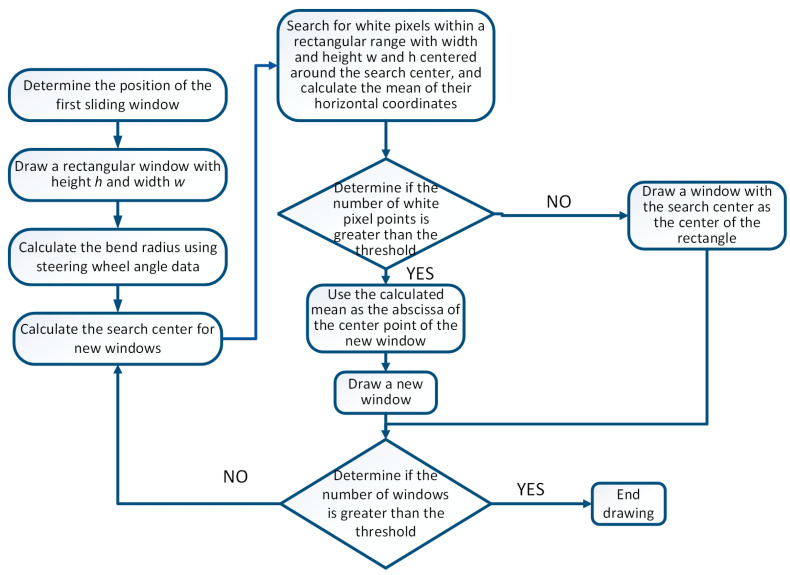
Flow chart of sliding window algorithm integrating steering wheel angle sensor.

The flow chart of the traditional sliding window algorithm for detecting lane lines was provided in the previous text. Due to the poor detection performance of the traditional sliding window algorithm in curves with large curvature or curves with dashed lane lines, this paper proposes a sliding window algorithm, which integrates steering wheel angle sensors to address this situation. The traditional sliding window algorithm uses the center of the previous window as the search center for the next window, while the sliding window algorithm integrating the steering wheel angle sensor will use the previous moment’s steering wheel angle data to find the search center for each new window, and it will search for white pixels near the search center. If the number of pixel points is greater than the threshold, the mean of the abscissa values of these points is used as the center of the new window. If it is not greater than the threshold, the search center is used as the center of the new window. Use the least squares method to fit the center points of these sliding windows to obtain lane lines. Finally, the lane line fitting map under normal viewing angle is obtained through inverse perspective transformation. When the data of the steering wheel angle sensor at the previous moment are zero, fusion is not performed. The specific flow chart is shown in Figure 11.

**Figure 12:**
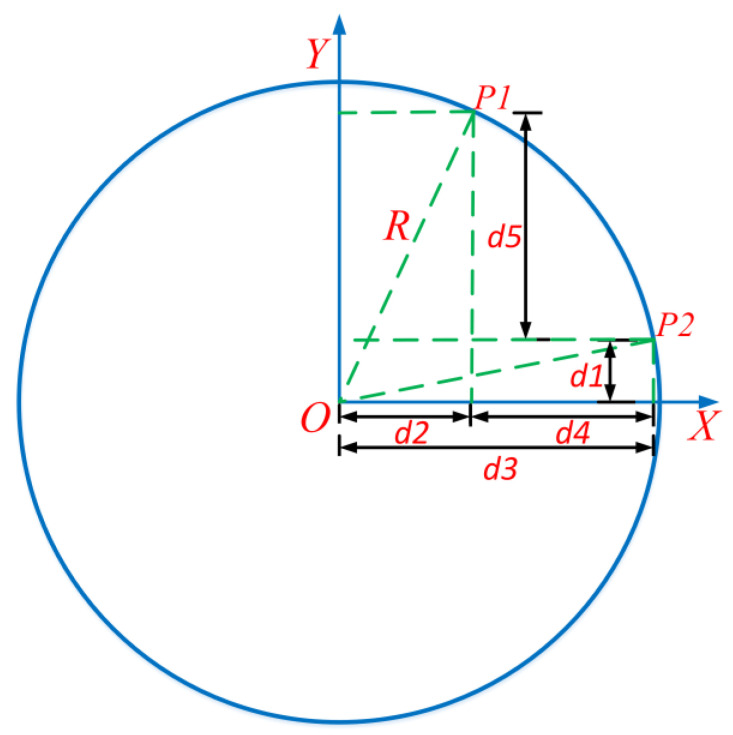
Schematic diagram for calculating lateral distance in curves.

**Figure 13:**
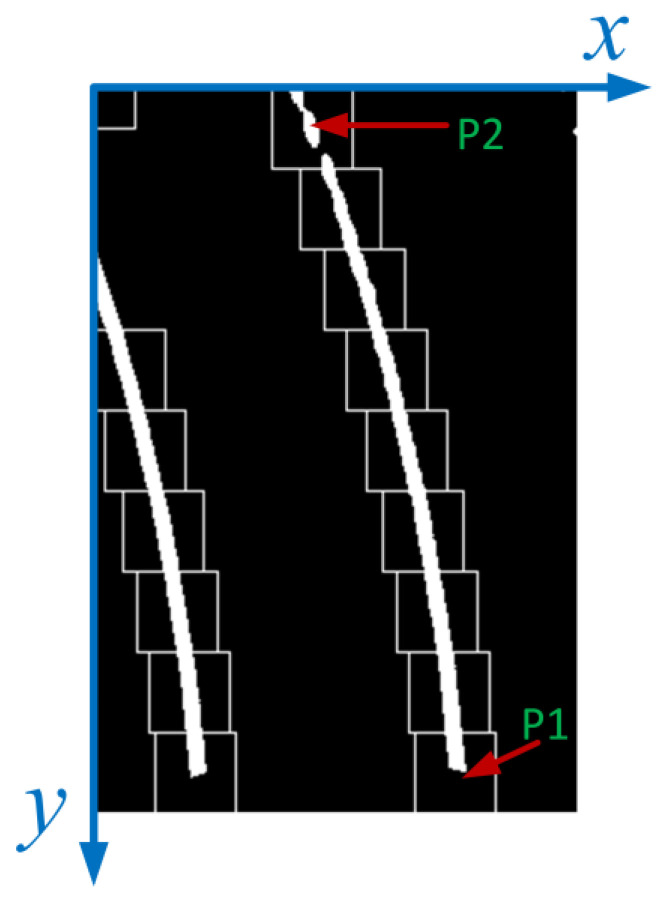
Schematic diagram of distance calculation between two points in aerial view.

From Figure 11, it can be seen that the most important aspect of the algorithm proposed in this article is the determination of the search center for each sliding window. Only when the search center is determined can white pixels be searched more accurately within the search range; when white pixels cannot be searched, the search center should be used as the rectangular center of the new sliding window. Therefore, we require the error between the search center and the actual center of the sliding window to be as small as possible. The schematic diagram for calculating the lateral distance between two points on the lane when the car is driving in a bend is shown in Figure 12. In Figure 12, there are two points *P*1 and *P*2 in the curve, and the lateral distance difference between *P*1 and *P*2 needs to be calculated. From Figure 12, it can be seen that the difference between *P*1 and *P*2 in the lateral distance is *d*4, *d*4 *= d*3 − *d*2. The calculation of *d*3 is related to the radius *R* and *d*1, while the calculation of *d*2 is related to *d*5, *d*1, and radius *R*. Therefore, to obtain *d*4, it is necessary to first calculate *d*1, *d*5, and radius *R*. The radius *R* can be calculated using the steering wheel angle sensor data and Formulas (3)–(5), with the key point being the calculation of *d*5 and *d*1. As shown in Figure 13, *d*5 can be calculated by using the pixel difference in the aerial view image. In the aerial view, *P*1 and *P*2 are the center points of the two sliding windows, and the width and height of the sliding windows are known. Therefore, the pixel difference of *P*1 and *P*2 in the y-direction of the aerial view image can be obtained, and then, the longitudinal distance ratio obtained in the previous article can be used to calculate the longitudinal distance difference between *P*1 and *P*2 in the real world.

In this algorithm, the center point position of each sliding window is referenced to the center point of the first sliding window. The turning radius of a car in this article refers to the turning radius of the rear axle of the vehicle. Therefore, in Figure 12, the value of d1 is the distance from the projection point of the center point of the first sliding window in the world coordinate system to the rear axle of the car. This value is fixed and can be obtained through measurement. The value obtained in the simulation software is 2.6 m. Therefore, the lateral difference between P1 and P2 points in the world coordinate system can be calculated. The pixel difference in the x-direction of P1 and P2 points in the pixel coordinate system can be obtained by the ratio of the lateral distance obtained in the previous text. Similarly, the horizontal pixel difference between the center point of other windows and the center point of the first window can also be obtained using similar calculation steps, as shown in Formulas (7)–(9).

(7) $$d_{yi}=\frac{y_{1}-y_{i}}{k_{y}}$$

$d_{yi}$ represents the longitudinal distance between the search center of the *i*-th sliding window (projected in the world coordinate system) and the center of the first sliding window (projected in the world coordinate system) in the world coordinate system. $y_{i}$ represents the y-coordinate value of the *i*-th sliding window search center in the pixel coordinate system. The longitudinal distance between the projection point of the *i*-th window search center in the world coordinate system and the rear axle of the vehicle needs to be added to 2.6 m. We set $D_{y1}$ = 2.6, $D_{yi}=d_{yi}+D_{y1}$, where $D_{yi}$ represents the longitudinal distance between the search center of the *i*-th sliding window (projected in the world coordinate system) and the rear axle of the vehicle.

(8) $$D_{xi}=\sqrt{R^{2}-D_{yi}^{2}}$$

In Formula (8), $D_{xi}$ is the horizontal coordinate value of the *i*-th window’s search center after being converted to the world coordinate system. *R* is the radius of the curve. What we want to calculate is the x-coordinate difference between the search center of each sliding window and the center of the first sliding window in the pixel coordinate system of the aerial view. The calculation method is shown in Formula (9).

(9) $$△x_{i}=(D_{xi}-D_{x1})\cdot k_{x}$$

In Formula (9), $∆x_{i}$ is the difference in the x-coordinate value between the search center of the *i*-th sliding window and the center of the first sliding window in the pixel coordinate system; $k_{x}$ is the ratio of lateral distance; $k_{x}$ was determined earlier.

One indicator to verify the effectiveness of the algorithm is the difference between the search center of each sliding window and its actual center in the pixel coordinate system. We set up curves with radii of 40 m, 60 m, and 80 m in the simulation software Prescan, with a lane width of 3 m and a vehicle speed of 15 m/s. Simulate the video and calculate the difference in x-coordinate values between the search center and the actual center of each window in different curves. The results are shown in Table 4, Table 5 and Table 6.

The curve with a radius of 40 m selects the sliding window of the right lane line, while the curve with a radius of 60 m and 80 m selects the sliding window of the left lane line. From Table 4, Table 5 and Table 6, it can be seen that the difference in x-coordinate values between the search center of the window and the actual center of the window is very small, and as the y-coordinate value of the window center gradually decreases, the difference will not diverge, still ensuring a small error. Therefore, when the lane lines in the curve are dashed, and some white pixels cannot be searched inside the sliding window, we can use the search center as the actual center of the sliding window. This ensures that the subsequent lane fitting process can be more closely aligned with the actual lane line.

To verify the accuracy of the algorithm proposed in this article, we conducted simulations and experiments. Using Prescan software to simulate the driving scene of a vehicle, obtain some parameter information of the vehicle and videos captured by a monocular camera during the driving process. Use OpenCV to process the video and obtain the lane line fitting of the vehicle during driving. Some parameter information of the vehicle is shown in Table 7.

**Figure 14:**
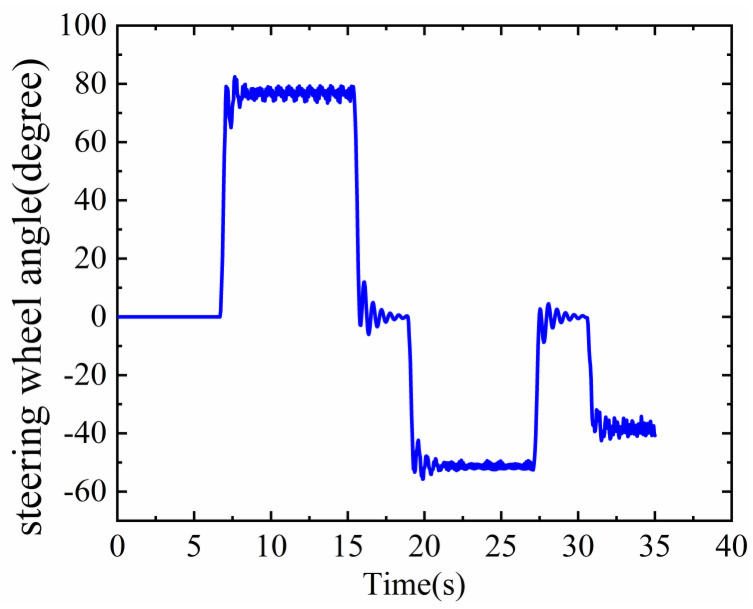
Steering wheel angle information.

In the simulation, the width of the lane is 2.5 m, and the driving speed is 15 m/s. The vehicle passed through curves with radii of 40 m, 60 m, and 80 m, respectively. The steering wheel angle information of the vehicle during driving is shown in Figure 14.

**Figure 15:**
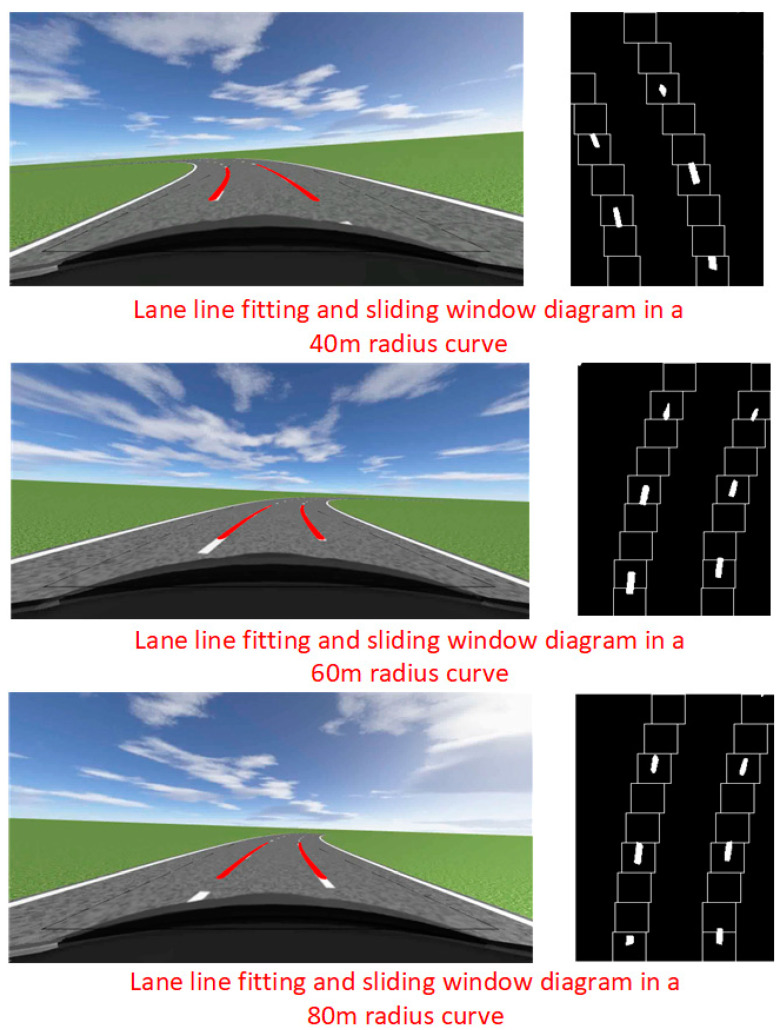
Fitting diagram and sliding window diagram of lane lines in curves.

The lane line fitting diagram of the vehicle in the curve is shown in Figure 15. From Figure 15, it can be seen that the sliding window algorithm integrating steering wheel angle sensors has quite good performance when vehicles are driving in curves with dashed lane lines. From the sliding window graph, it can also be seen that in the parts without white pixels, the sliding window can still track the lane line very well. Compared to the traditional sliding window algorithm, which cannot track lane lines well in high curvature curves and dashed curves, the improved sliding window algorithm proposed in this article effectively solves this defect. This proves the effectiveness of the algorithm proposed in this article.

## 4. Using Binocular Cameras to Assist in Locating the First Sliding Window

In the previous article, we used the pixel statistics method to determine the location of the search center of the first window (the search center of the sliding window at the bottom). Since we intercepted 1/5 of the aerial view for pixel statistics, when the vehicle enters the dotted lane, the situation shown in Figure 6 will occur. At this point, the method of pixel statistics cannot determine the first sliding window. From Table 4, Table 5 and Table 6, it can be seen that the centers of the new windows are all based on the center of the first window as a reference, so it is very important to accurately locate the center of the first window. We propose a method for obtaining the center of the first sliding window using a binocular camera in response to the inability of pixel statistics to locate the position of the first sliding window. The binocular camera is installed below the vehicle’s rear-view mirror, so its perspective can compensate for the shortcomings of pixel statistics. The speed of processing binocular camera images is slow, so it can only be used as an aid, and the positioning of binocular cameras is only used as a standard when pixel statistics fail. The specific implementation method is to install the binocular camera under the two rear-view mirrors of the vehicle, take an aerial view of the lane line, and measure the distance between the two sides of the vehicle and the lane line. Convert the distance measured by a binocular camera into a pixel coordinate system through distance ratio. The principle of using a binocular camera to locate the first sliding window is shown in Formula (10).

(10) $$\begin{array}{l} x_{l}=m-(s_{l}+d_{l})\cdot k_{x}\\ x_{r}=m+(s_{r}+d_{r})\cdot k_{x} \end{array}$$

**Figure 16:**
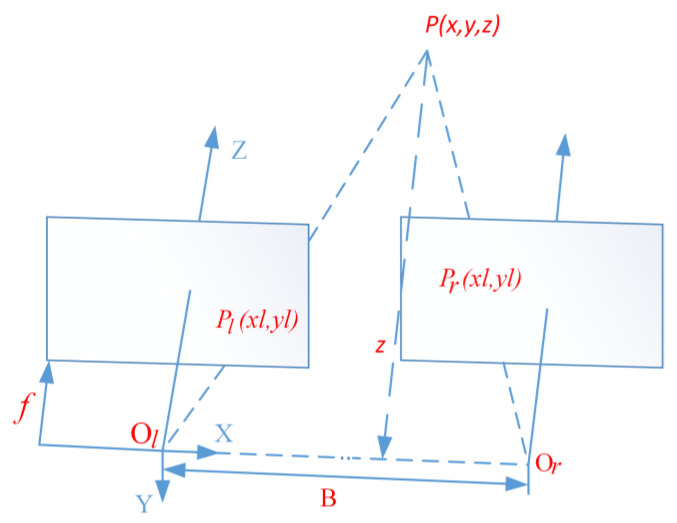
Schematic diagram of binocular camera ranging.

In Formula (10), $x_{l}$ is the abscissa value of the first sliding window center of the left lane line in the aerial view pixel coordinate system, and $x_{r}$ is the abscissa value of the first sliding window center of the right lane line in the aerial view pixel coordinate system. *m* is half of the width of the aerial view image. For example, the width of the aerial view image in the previous article is 240, so the *m* here is 120. $s_{l}$ is the distance from the left binocular camera to the left lane line, and $s_{r}$ is the distance from the right binocular camera to the right lane line. $s_{l}$ and $s_{r}$ are both obtained through binocular camera ranging. $d_{l}$ is the distance between the optical axis of the left binocular camera and the longitudinal axis of the vehicle, while $d_{r}$ is the distance between the optical axis of the right binocular camera and the longitudinal axis of the vehicle. The units of $s_{l}$, $s_{r}$, $d_{l}$, and $d_{r}$ are all in meters. $k_{x}$ is the ratio of the horizontal pixel distance in the aerial view to the horizontal distance in the world coordinate system, and the method to obtain it was given in the previous article. The basic principle of binocular camera ranging is to use two cameras to complete binocular image acquisition of the target in front of the lens. Due to the difference in shooting angle and position between the left and right cameras during image acquisition, there is a certain disparity between the two images of the target object obtained by the camera at the same time. Based on the disparity, the spatial coordinates of the target object are obtained [21]. The ranging principle of a binocular camera is shown in Figure 16.

In Figure 16, $O_{l}$ and $O_{r}$ are the optical center positions of the two cameras. The length of the connection between $O_{l}$ and $O_{r}$ is the baseline distance; *B*, *Z* is the optical axis of the left camera; and the left and right cameras are parallel. If there is a point *P* (x, y, z) in space, the projection points on the left and right camera images are $P_{l}(x_{l},y_{l})$ and $P_{r}(x_{r},y_{r})$. When the installation of the left and right cameras is ideal, there is $y_{l}=y_{r}=y^{\prime }$. Formula (11) can be obtained from similar triangles.

(11) $$\{\begin{array}{c} x_{l}=f\frac{x}{z}\\ x_{r}=f\frac{B-x}{z}\\ y^{\prime }=f\frac{y}{z} \end{array}$$

By transforming the formula above, a coordinate formula that can solve spatial point P can be derived, as shown in Formula (12).

(12) $$\{\begin{array}{c} x=\frac{B\cdot x_{l}}{x_{l}-x_{r}}=\frac{B\cdot x_{l}}{D}\\ y=\frac{B\cdot y^{\prime }}{x_{l}-x_{r}}=\frac{B\cdot y^{\prime }}{D}\\ Z=\frac{B\cdot f}{x_{l}-x_{r}}=\frac{B\cdot f}{D} \end{array}$$

In Formula (12), $D=x_{l}-x_{r}$ is referred to as the parallax between the two images captured by the left and right cameras. From the above equation, it can be seen that in order to use a binocular camera to determine the coordinates of a spatial point, it is necessary to obtain the baseline distance *B* of the left and right cameras, the parallax *D* of the left and right images, and the focal length *f* of the camera. Among them, *B* and *f* are obtained through camera calibration. In this article, the Zhang Zhengyou calibration method [22] is used to calibrate the binocular camera, and the disparity *D* is obtained through stereo matching. This article uses the block matching (BM) algorithm in OpenCV for stereo matching, which has the advantage of fast algorithm speed and meets our requirements for using it on driving vehicles.

**Figure 17:**
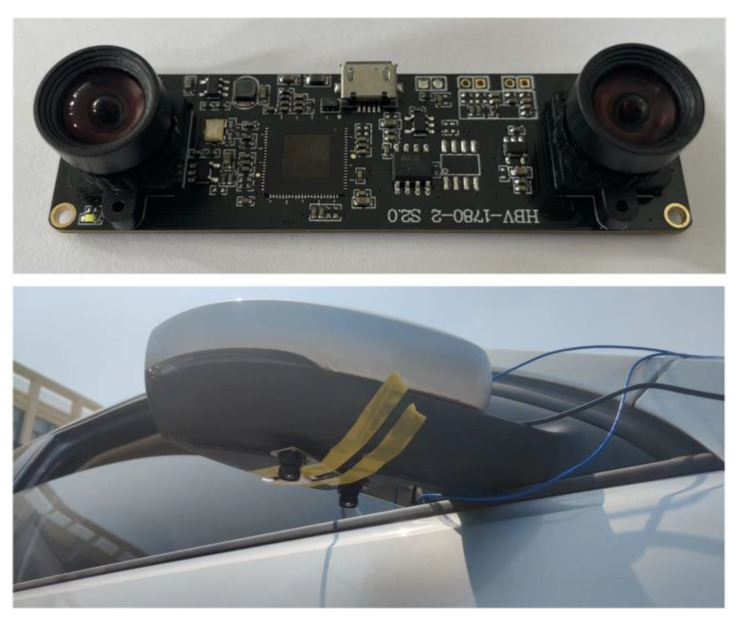
Installation diagram of binocular camera.

We conducted experiments on the ranging of binocular cameras. We divide the ranging experiments of binocular cameras into static ranging and dynamic ranging. The binocular camera we use and the installation diagram of the camera are shown in Figure 17.

**Figure 18:**
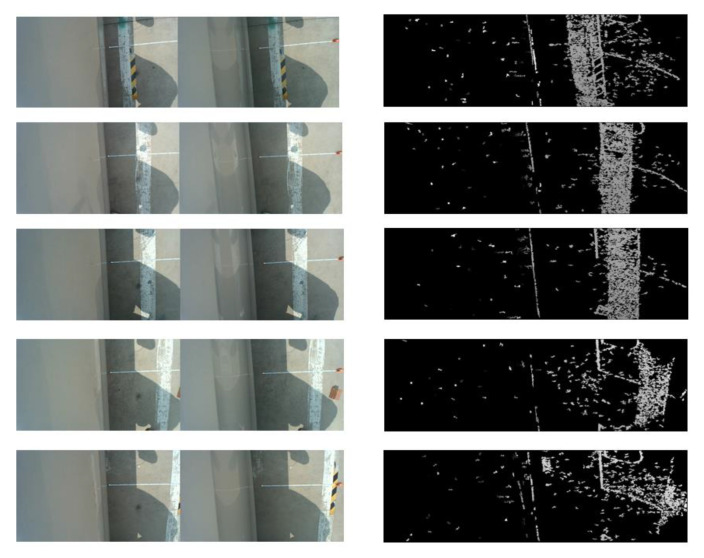
Static ranging experimental diagram.

The static ranging of binocular cameras is the process of shooting and ranging the lane line when the vehicle is stationary beside the lane line. The selection of lane line pixel points is manual, and the main purpose of static ranging is to verify whether the accuracy of binocular camera ranging meets the requirements of this article. The static ranging experiment image of the binocular camera is shown in Figure 18. The error results of distance measurement are shown in Table 8.

**Figure 19:**
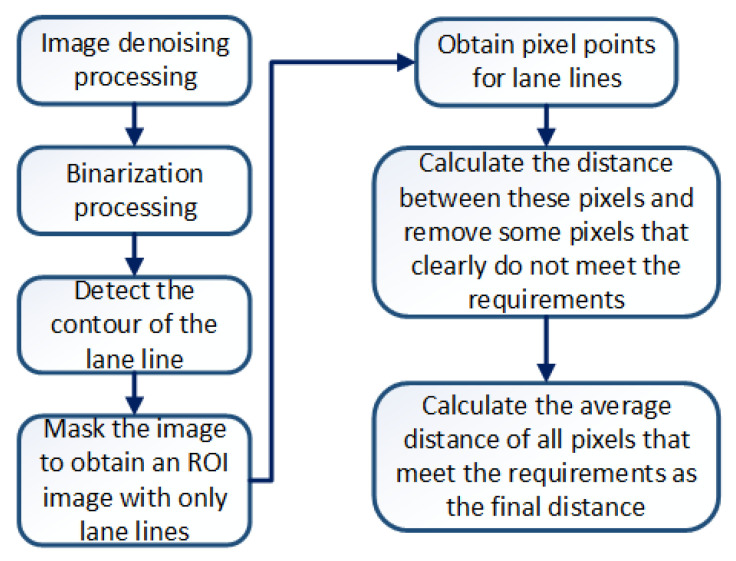
Dynamic ranging flowchart.

The left side of Figure 18 is the original image taken by a binocular camera, and the right side is the disparity map. The measured distances are all based on the optical center of the left camera of the binocular camera as the reference origin. In static ranging experiments, the error between the measured distance and the actual distance is on the millimeter level, with a maximum error of 19 mm, which can meet the accuracy of ranging. With dynamic ranging, it is necessary to first identify the lane line and then obtain the pixel information of the lane line. Unlike static ranging, the pixel points of lane lines are extracted through algorithms during dynamic ranging. We determine the pixel information of points on the lane line by extracting the contour of the lane line [23]. The results of dynamic ranging are shown in Table 9. The specific process of dynamic ranging is shown in Figure 19.

**Figure 20:**
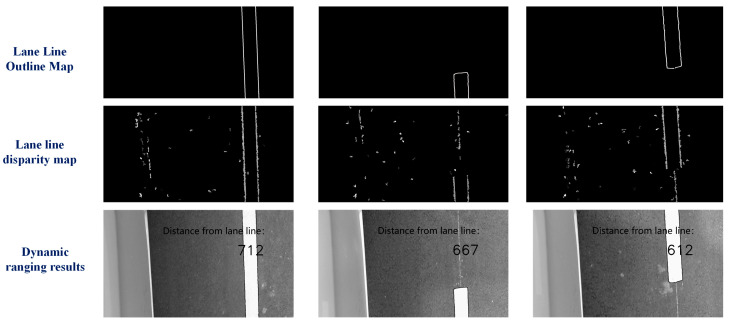
Results of binocular dynamic ranging.

The real-time image captured during dynamic ranging using a binocular camera is shown in Figure 20.

From Figure 20, it can be seen that lane lines can be well detected through contour extraction during vehicle driving. The distance measurement results in Table 9 show that although the error is greater than that in the static state, the error results are still within an acceptable range. Taking the maximum error of 23 mm as an example, based on the lateral distance ratio obtained from the previous experiment, the pixel error in the x-direction of the pixel coordinate system is only one pixel, which is very small. Therefore, using binocular cameras to assist in locating the first sliding window is feasible.

## 5. Experiment on Sliding Window Algorithm Integrating Steering Wheel Angle Sensor and Binocular Camera

**Figure 21:**
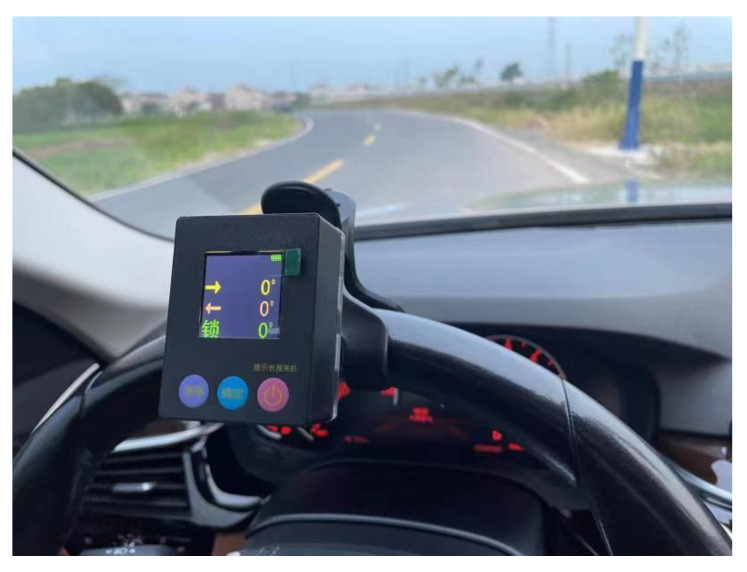
Installation and calibration of steering wheel angle sensor.

We conducted real-world experiments on the improved sliding window algorithm, and the vehicle parameters are shown in Table 10. Install monocular cameras on the vehicle windshield, with two monocular cameras installed below the rear-view mirrors on both sides of the vehicle. The monocular camera coincides with the longitudinal axis of the vehicle, while the binocular camera is perpendicular to the longitudinal axis of the vehicle. Real-time measurement of the steering wheel angle of the vehicle is achieved through an angle sensor. The installation and calibration of the steering wheel angle sensor are shown in Figure 21.

**Figure 22:**
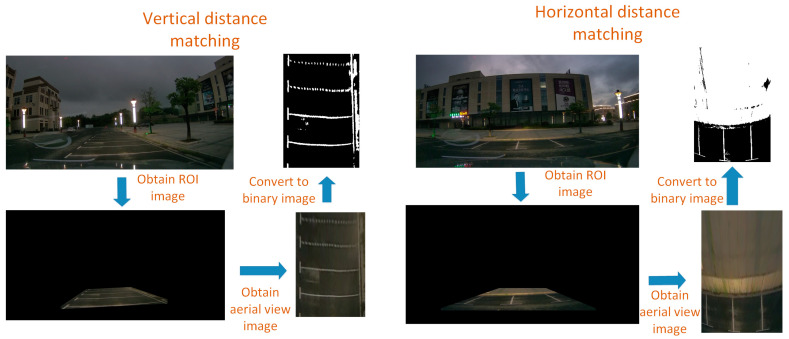
Obtaining distance ratio.

The actual distance ratio of pixel distance in the aerial view is determined by shooting the grid lines on the ground. The spacing between grid lines in the world coordinate system is 2.5 m. The process of obtaining the distance ratio is shown in Figure 22.

The width and height of the aerial view are 240, 360, respectively. The width and height of the original image are 640, 360, respectively. The four points of the perspective transformation taken in the original image are shown in Table 11. The four points after the perspective transformation are a (0,0), b (0,360), c (240,360), and d (240,0). According to experimental data, one meter of vertical distance in the world coordinate system has 36 pixel values in the pixel coordinate system, while one meter of horizontal distance in the world coordinate system has 34.4 pixel values in the pixel coordinate system.

**Figure 23:**
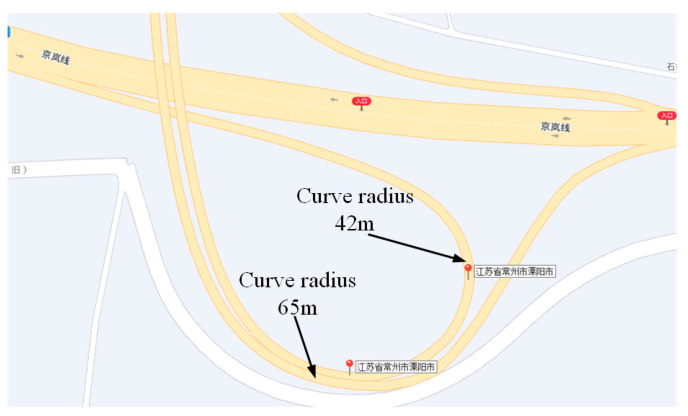
Map of the curves that the vehicle passed through.

We conducted real vehicle experiments on curves with radii of 65 m and 42 m, and the curves the vehicle passed through are shown in Figure 23.

**Figure 24:**
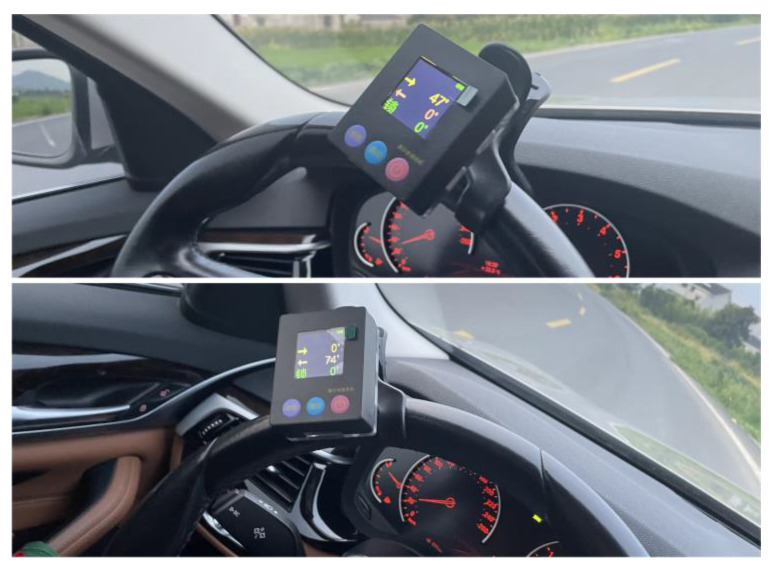
Steering wheel angle information in bends.

The steering wheel angle data in the curve are shown in Figure 24, and the vehicle’s speed in both curves is 30 km/h.

**Figure 25:**
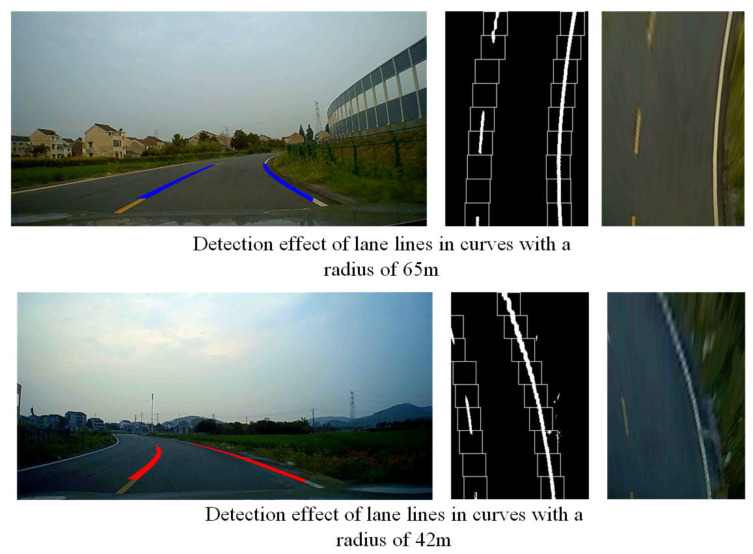
Lane recognition results in curves.

The vehicle’s speed in both curves is 30 km/h. Through actual measurement, the distance from the projection point of the first sliding window center in the world coordinate system to the rear axle of the vehicle is 4.8 m. The lane line recognition result of the vehicle in the curve is shown in Figure 25, which also includes the aerial view of the road in the curve and the tracking effect of the sliding window on the lane line.

From Figure 25, it can be seen that in actual vehicle experiments, the algorithm proposed in this paper can effectively detect lane lines in curves, proving that the algorithm also performs well in practical applications.

## 6. Discussion and Conclusions

This article proposes a sliding window algorithm integrating steering wheel angle sensor and binocular camera. Integrating the steering wheel angle information from the previous moment into the sliding window algorithm for the next moment, the binocular camera is used to assist in locating the first sliding window. The traditional sliding window algorithm has poor recognition and tracking performance for lane lines in curves with large curvature or dashed lane lines. The algorithm proposed in this paper effectively solves this problem, and the assistance of binocular cameras improves the robustness of the algorithm. The effectiveness of the new algorithm proposed in this article was verified through simulation and experiments. This is due to the fact that the lane detection algorithm used in this article is essentially based on brightness threshold segmentation in image processing. Therefore, the performance of the algorithm may vary under different weather and lighting conditions. In subsequent research, weather and lighting conditions can be emphasized, and different thresholds can be set for different lighting conditions to make the algorithm more robust. We can also use deep-learning methods to create a dataset of lane lines.

## Figures and Tables

**Table 1:** Pixel coordinate values of each point in the ROI area.

	X-Coordinate Value	Y-Coordinate Value
A	640/10 × 4.4	360/5 × 2.75
B	640/8 × 2	360/8 × 7
C	640/8 × 6	360/8 × 7
D	640/10 × 5.6	360/5 × 2.75

**Table 2:** Relationship between horizontal distance and actual distance in aerial view.

Actual Lane Width (m)	Lane Width in Aerial View (Pixel)	Ratio (Pixel/m)
3	136	45.3
3.5	156	44.6
4	178	44.5
4.5	199	44.2

**Table 3:** Relationship between longitudinal distance and actual distance in aerial view.

Actual Lane Width (m)	Lane Width in Aerial View (Pixel)	Ratio (Pixel/m)
3	92	30.7
3.5	108	30.9
4	124	31
4.5	138	30.7

**Table 4:** The x-coordinate value error between the search center of the window and the actual center of the window in a bend with a radius of 40 m.

Window Number	Window Center Point Coordinates	The Search Center Coordinates of the Window	The Difference in X-Coordinate Value between the Actual Center of the New Window and the Center of the First Window (Pixel)	The Difference in X-Coordinate Value between the Search Center of the New Window and the Center of the First Window (Pixel)	Error (Pixel)
1	(187,340)	-	-	-	-
2	(183,300)	(182,300)	4	5	1
3	(176,260)	(175,260)	11	12	1
4	(167,220)	(167,220)	20	20	0
5	(156,180)	(156,180)	31	31	1
6	(143,140)	(144,140)	44	43	1
7	(129,100)	(129,100)	58	58	0
8	(113,60)	(112,60)	74	75	1
9	(97,20)	(93,20)	90	94	4

**Table 5:** The x-coordinate value error between the search center of the window and the actual center of the window in a bend with a radius of 60 m.

Window Number	Window Center Point Coordinates	The Search Center Coordinates of the Window	The Difference in X-Coordinate Value between the Actual Center of the New Window and the Center of the First Window (Pixel)	The Difference in X-Coordinate Value between the Search Center of the New Window and the Center of the First Window (Pixel)	Error (Pixel)
1	(51,340)	-	-	-	-
2	(54,300)	(55,300)	3	4	1
3	(58,260)	(59,260)	7	8	1
4	(64,220)	(65,220)	13	14	1
5	(71,180)	(72,180)	20	21	1
6	(80,140)	(80,140)	29	29	0
7	(89,100)	(89,100)	38	38	0
8	(100,60)	(100,60)	49	49	0
9	(113,20)	(112,20)	62	61	1

**Table 6:** The x-coordinate value error between the search center of the window and the actual center of the window in a bend with a radius of 80 m.

Window Number	Window Center Point Coordinates	The Search Center Coordinates of the Window	The Difference in X-Coordinate Value between the Actual Center of the New Window and the Center of the First Window (Pixel)	The Difference in X-Coordinate Value between the Search Center of the New Window and the Center of the first Window (Pixel)	Error (Pixel)
1	(53,340)	-	-	-	-
2	(55,300)	(56,300)	2	3	1
3	(58,260)	(59,260)	5	6	1
4	(62,220)	(63,220)	9	10	1
5	(68,180)	(69,180)	15	16	1
6	(73,140)	(75,140)	20	22	2
7	(81,100)	(82,100)	28	29	1
8	(89,60)	(90,60)	36	37	1
9	(98,20)	(99,20)	45	46	1

**Table 7:** Vehicle parameter information.

Name of Vehicle Parameters	Parameter Value
Total lateral stiffness of the front wheels (N/rad)	−80,000
Total lateral stiffness of the rear wheels (N/rad)	−67,041
Front axle wheelbase (m)	0.95
Rear axle wheelbase (m)	1.42
Wheelbase (m)	2.37
Body mass (kg)	1005
Steering ratio	20

**Table 8:** Static ranging data of binocular cameras.

Serial Number	Actual Distance (mm)	Measuring Distance of Binocular Camera (mm)	Error (mm)
1	159	163	4
2	339	330	9
3	342	345	3
4	522	529	7
5	670	651	19

**Table 9:** The error of dynamic ranging with binocular cameras.

Serial Number	Actual Distance (mm)	Measuring Distance of Binocular Camera (mm)	Error (mm)
1	727	712	10
2	650	667	17
3	635	612	23

**Table 10:** Parameters of the experimental vehicle.

Parameter Name	Parameter Value
Total lateral deflection stiffness of the front wheel (N/rad)	−157,126
Total lateral deflection stiffness of the rear wheel (N/rad)	−136,116
Front axle wheelbase (m)	1.368
Rear axle wheelbase (m)	1.742
Wheelbase (m)	3.11
Body mass (kg)	1660
Steering ratio	17

**Table 11:** Value of each point in perspective transformation.

	X-Coordinate Value	Y-Coordinate Value
A	640/10 × 3	360/10 × 7
B	640/10 × 1.6	360/10 × 8
C	640/10 × 8.4	360/10 × 8
D	640/10 × 7	360/10 × 7

## Data Availability

The data used to support the findings of this study are included within the article.
